# The *rgg*_*0182 *_gene encodes a transcriptional regulator required for the full *Streptococcus thermophilus *LMG18311 thermal adaptation

**DOI:** 10.1186/1471-2180-11-223

**Published:** 2011-10-07

**Authors:** Romain Henry, Emmanuelle Bruneau, Rozenn Gardan, Stéphane Bertin, Betty Fleuchot, Bernard Decaris, Nathalie Leblond-Bourget

**Affiliations:** 1 INRA, UMR1128 Génétique et Microbiologie, F-54506 Vandœuvre-lès-Nancy Cedex, France; 2 Université de Lorraine, UMR1128 Génétique et Microbiologie, F-54506 Vandœuvre-lès-Nancy Cedex, France; 3 INRA, UMR1319 MICALIS, Equipe Peptides et Communication Bactérienne, F-78352 Jouy en Josas, France

## Abstract

**Background:**

*Streptococcus thermophilus *is an important starter strain for the production of yogurt and cheeses. The analysis of sequenced genomes of four strains of *S. thermophilus *indicates that they contain several genes of the *rgg *familly potentially encoding transcriptional regulators. Some of the Rgg proteins are known to be involved in bacterial stress adaptation.

**Results:**

In this study, we demonstrated that *Streptococcus thermophilus *thermal stress adaptation required the *rgg*_*0182 *_gene which transcription depends on the culture medium and the growth temperature. This gene encoded a protein showing similarity with members of the Rgg family transcriptional regulator. Our data confirmed that Rgg_0182 _is a transcriptional regulator controlling the expression of its neighboring genes as well as chaperones and proteases encoding genes. Therefore, analysis of a Δ*rgg*_*0182 *_mutant revealed that this protein played a role in the heat shock adaptation of *Streptococcus thermophilus *LMG18311.

**Conclusions:**

These data showed the importance of the Rgg_0182 _transcriptional regulator on the survival of *S. thermophilus *during dairy processes and more specifically during changes in temperature.

## Background

The species *Streptococcus thermophilus *is a Lactic Acid Bacterium (LAB) used as a starter of fermentation in yogurt and cheese production. In nature and during dairy fermentation processes, *S. thermophilus *is subjected to sudden changes in its environment and its industrial performance is conditioned by its ability to successfully adapt to harsh conditions. To survive, like many other bacteria, this species must develop appropriate physiological responses by modifying gene expression appropriately.

One of the stresses, that *S. thermophilus *commonly encounters, is the modification of the temperature. For instance, during the production of dairy products, temperature shifts are applied to regulate the bacterial growth and, thus, control the lactic acid production [1]. *S. thermophilus *survival against thermal stress is conditioned by its ability to sense and quickly adapt its physiology mainly by the synthesis of adequate proteins at the right moment. For example, adaptation of *S. thermophilus *to a lowering of temperature required the synthesis of a set of chaperones called cold shock proteins (Csp) that is strongly induced in response to a rapid decrease in growth temperature [2,3]. As in other Gram positive bacteria, *S. thermophilus *also responds to thermal stress by synthesizing a conserved set of heat-shock proteins (Hsp), including both chaperones and proteases [4]. Their role during heat stress is to rescue, or to scavenge, heat-denatured proteins. In LAB, the adaptation to heat shock required the two negative transcriptional factors HrcA [5] and CtsR [6] which negatively control the expression of major molecular chaperones (DnaK, DnaJ, GroEL and GroES) [7] and proteases (Clp family) [6], respectively. Furthermore, Zotta *et al*. (2009) have shown the involvement of the HrcA and CtsR proteins in the heat stress response of *S. thermophilus *Sfi39 [8]. Apart from these data, little is known about the network of regulation controlling *S. thermophilus *adaptation to temperature changes.

Among bacterial transcriptional regulators is the wide conserved family of Rgg regulators encoded by genes, exclusively found in the order of *Lactobacillales *and the family *Listeriaceae *[9]. Rgg regulators act by binding to the promoter region of their target genes [10-13]. At their N-terminal end, they carry a Helix-Turn-Helix (HTH) XRE DNA-binding domain demonstrated to be important for their activity as transcriptional regulators [14]. They are positive regulator [15,16] or act both as activator and repressor [17,18]. Most of the Rgg regulators control the transcription of their neighboring genes [9,16,19,20]. However, Rgg from *S. pyogenes *NZ131, *S. agalactiae *NEM316 or *S. suis *SS2 are considered as global regulators since controlling highly diverse genes scattered on the genome [12,13,21,22]. In these cases, Rgg proteins are involved in a network of regulation and modulate the expression of other transcriptional regulators, including several two-component regulatory systems, which are important in the transcriptional response to changing environments [12,13,21]. Several Rgg proteins contribute to bacterial stress response. For instance, the Rgg protein of *Lactocccus lactis*, also known as GadR, is associated with glutamate-dependent acid tolerance [15]. Within *Streptococcus*, several Rgg proteins have been involved in oxidative- and/or to thermal-stress responses [23-25].

The high number of *rgg *genes observed in the genomes of *S. thermophilus *strains (7 in strains LMG18311 and CNRZ1066, 6 in LMD-9 and 5 in ND03) [26-28] suggests that their acquisition and their preservation are advantageous for *S. thermophilus*. However, the involvement of these genes in *S. thermophilus *LMG18311 stress response is still hypothetic and none of the 7 *rgg *genes of LMG18311 has been studied at the molecular level. To determine whether any of the *rgg *genes of *S. thermophilus *LMG18311 are involved in adaptation to changes in environmental conditions, Δ*rgg *deletion mutant was constructed and its tolerance to different stresses was tested. In this study, we demonstrate that (i) the transcription of *rgg*_*0182 *_gene from *S. thermophilus *LMG18311 is influenced by culture medium and growth temperature, (ii) Rgg_0182 _is a transcriptional regulator that modulate not only the transcription of its proximal target genes but is also involved in the network of regulation of the transcription of genes coding chaperones and proteases, (iii) this gene is involved in heat shock response.

## Results

### Analysis of the *rgg*_*0182 *_locus

**Figure 1:**
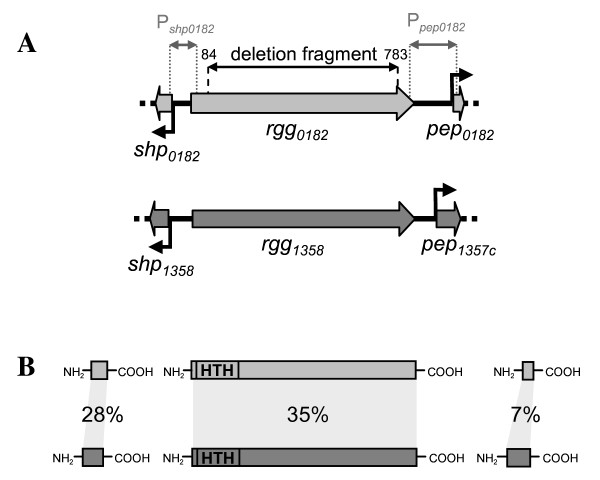
**Schematic representation of the *rgg***_***0182 ***_**and *rgg***_***1358 ***_**loci (A) and of the corresponding proteins (B)**. Although the *rgg*_*0182 *_and *rgg*_*1358 *_loci present analogies (A), they encoded distinct proteins (B). Numbers in panel A indicate the position of nucleotides, with the +1 position being that of the first nucleotide of the *rgg*_*0182 *_gene. The "deletion fragment" corresponds to the deleted portion of the *rgg*_*0182 *_gene in the Δ*rgg*_*0182 *_mutant. The broken arrows indicate the promoters. P*shp*_*0182 *_and P*pep*_*0182*_materialized the position of the 126 bp and 165 bp PCR fragment respectively used in EMSA. In panel B, amino acids sequence identities are indicated in percent. HTH indicated the Helix-Turn-Helix-XRE motif.

The *rgg*_*0182 *_gene corresponds to the *stu0182 *gene of the complete genome sequence of *S. thermophilus *LMG18311 [26]. However, the sequencing of the *rgg*_*0182 *_gene (GenBank Accession JF699754) followed by its sequence comparison with the *stu0182 *gene revealed that this latter contained sequencing discrepancies (substitutions in positions 44, 46, 50, 579 and 681 compared to the *rgg*_*0182 *_sequence). The *rgg*_*0182 *_gene (864 bp) potentially encodes a protein of 288 amino acids with a predicted molecular mass of 35.6 kDa. This protein exhibited an identity of about 30% with other streptococcal proteins belonging to the Rgg family of transcriptional regulators and 35% identity (e-value = 8e^-48^) with Rgg_1358 _from *S. thermophilus *LMD-9 which was recently shown to be involved in a quorum sensing (QS) mechanism [9]. Rgg_0182 _contained a HTH-XRE motif from amino acid 11 to 67 typical of Rgg regulators and a Rgg-C-terminal motif from amino acid 70 to 288 (Figure 1). Therefore, the *rgg*_*0182 *_gene was predicted to encode a transcriptional regulator.

The gene *rgg*_*0182 *_was surrounded by two ORFs (Figure 1), not annotated in the genome of the strain LMG18311, but revealed using the software bactgeneSHOW designed for small-gene detection [29]. Indeed, upstream of the *rgg*_*0182 *_gene was the *shp*_*0182 *_gene (63 nucleotides long), potentially encoding a small hydrophobic peptide belonging to the group I of the SHP family [9]. Downstream of *rgg*_*0182 *_was the *pep*_*0182 *_gene (42 nucleotides long), encoding a small peptide with no similarity with peptides found in databases. Although, the genetic organization of the *rgg*_*0182 *_locus was similar to that of the *rgg*_*1358 *_of the LMD-9 strain from *S. thermophilus*, these two loci were distinct as illustrated by the low sequence identity between the proteins encoded by them (Figure 1). The two *shp *genes were classified in two distinct groups from the SHP family [9]. Finally, the *rgg*_*0182 *_locus and its flanking genes were also found in the genome of CNRZ1066 strain but missing in the genome of ND03 and LMD-9 strains.

### Transcription analysis of the *rgg*_0182 _gene

In the literature, studies of *rgg *genes transcription are scarce. Indeed, only the *ropB *transcription from *Streptococcus pyogenes *has been studied [10]. Thus, it was of interest to determine whether transcription of *rgg *was constitutive or not. To do so, the *rgg*_*0182 *_transcription was studied by qPCR from cells cultivated in LM17 or CDM medium at 30 or 42°C. In all qPCR experiments, the values were normalized to the expression of the *ldh *gene encoding the lactate dehydrogenase. This gene was considered as a relevant reference since it was demonstrated to be constitutively expressed in all tested conditions (data not shown). The qPCR experiments were realized from three independent RNA extracts and done in duplicate.

**Figure 2:**
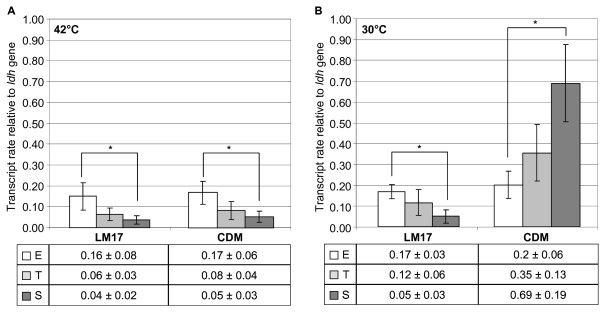
**Relative *rgg***_***0182 ***_**gene transcript level from *S. thermophilus *LMG18311 cells, grown at 42°C (A) and at 30°C (B)**. Total RNAs from the wild type strain were extracted from exponential (E, white bars), transition (T, light gray bars) and stationary (S, dark gray bars) phase cells. Data are presented as the mean +/- standard deviation from three independent experiments performed in duplicate. Student's *t *test: *, p < 0.001.

Figure 2A showed the relative transcription levels of the *rgg*_*0182 *_gene in the LMG18311 strain. When cultivated at 42°C in LM17 medium, the wild-type strain showed a significant decrease in its *rgg*_*0182 *_mRNA levels during growth. Indeed, the *rgg*_*0182 *_mRNA level was highest in the exponential phase (0.16 +/- 0.08) and was down-regulated 4-fold in stationary phase (p = 0.01). Similar results were obtained in CDM medium at 42°C where the transcription of *rgg*_*0182 *_was found to be more than 3-fold higher in the exponential phase than in stationary phase (p < 0.001). Whatever the medium tested, the transcription of *rgg*_*0182 *_was found to be growth-phases dependent.

We then investigated whether *rgg*_*0182 *_was transcribed at other temperatures and chose to work at 30°C, temperature at which *S. thermophilus *can be exposed during industrial processes (Figure 2B). When cells were cultivated at 30°C in LM17, the profile of *rgg*_*0182 *_transcripts was similar with that observed at 42°C. In contrast when cells were grown at 30°C in CDM medium, an increase of *rgg*_*0182 *_transcription was observed during the growth, i.e. the *rgg*_*0182 *_mRNA level was more than 3-fold higher (p < 0.001) in stationary phase than in exponential phase. The *rgg*_*0182 *_transcripts level of stationary phase cells grown in CDM medium was 14-fold higher (p < 0.001) at 30 than at 42°C indicating it was on the influence of the growth temperature. Taken together, these results revealed that the kinetics of *rgg*_*0182 *_transcription was medium and temperature dependent and that the transcript level of *rgg*_*0182 *_was the highest in stationary phase cells cultivated in CDM at 30°C.

### Effects of the Rgg_0182 _protein on the transcription of its flanking genes

**Figure 3:**
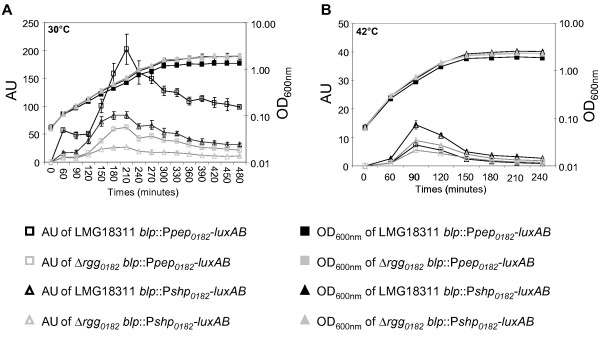
**Luciferase activity and growth of the LMG18311 and the Δ*rgg***_***0182 ***_**strains containing the P**_***shp0182***_**-*luxAB *and P**_***pep0182***_**-*luxAB *transcriptional fusions, in CDM medium**. The expression of the fusions was followed in strains cultivated in CDM medium, at 30°C (A) or at 42°C (B). Data are presented as the mean +/- standard deviation of three independent experiments. AU: luminescence arbitrary units normalized against the OD_600nm _of the cultures.

Data from the literature indicate that several products of *rgg *genes regulate adjacent genes [9,16,19,20]. To determine whether the product of the *rgg*_*0182 *_gene was involved in the transcriptional regulation of its flanking genes, we designed primers and used them in qPCR to measure the level of transcription of the *shp*_*0182 *_and *pep*_*0182 *_genes. However, the qPCR failed probably because of the small size of the *pep*_*0182 *_and *shp*_*0182 *_genes that did not allow the design of effective primers. As an alternative strategy, the activity of the *pep*_*0182 *_and *shp*_*0182 *_promoters was studied with transcriptional fusion in a wild-type and a Δ*rgg*_*0182 *_background. To do so, plasmids carrying transcriptional fusions coupling the intergenic region of each flanking gene to a *luxAB*-reporter fusion were constructed and named pGICB004::P_*pep0182 *_and pGICB004::P_*shp0182*_, as well as the Δ*rgg*_*0182 *_strain carrying a chromosomal deletion of the *rgg*_*0182 *_gene. Both plasmids were integrated in the wild-type or Δ*rgg*_*0182 *_mutant chromosome. Relative levels of activity of the *pep*_*0182 *_and *shp*_*0182 *_promoters were determined in both strains either grown in LM17 or CDM (Figure 3), at 30 or 42°C. Whatever the conditions, no significant difference in the growth rate or yield was observed between the wild type and the mutant. In LM17 at 30 and 42°C, almost no luciferase activity was detected with both promoters in the wild type or the Δ*rgg*_*0182 *_background (data not shown). This suggests that the promoters P_*pep0182 *_and P_*shp0182 *_are not active in these experimental conditions. In contrast in CDM medium, for both promoters in a wild type background, a luciferase activity was detected at 30°C and 42°C (Figure 3). Nevertheless, the maximum of P_*pep0182*_*-luxAB *and P_*shp0182*_*-luxAB *activity were 28- and 6-fold higher (p < 0.001) at 30°C than at 42°C, respectively. In addition, the level of activity of the P_*pep0182*_*-luxAB *and P_*shp0182*_*-luxAB *fusions differed between the wild-type and the mutant strains. Indeed, in cells cultivated in CDM at 30°C, in the Δ*rgg*_*0182 *_mutant the P_*pep0182*_*-luxAB *and the *P*_*shp0182*_*-luxAB *showed both a maximum activity that was 3-fold lower (p < 0.001) than in the LMG18311 strain (Figure 3). These results demonstrated that *rgg*_*0182 *_played a role in the regulation of the transcription of both P_*pep0182*_*-luxAB *and P_*shp0182*_*-luxAB *fusions and supported the hypothesis that Rgg_0182 _may, directly or not, regulate the transcription of *pep*_*0182 *_and *shp*_*0182 *_genes. Moreover, the growth medium, as described above and by Ibrahim *et al*. (2007b), and, in an original way, the temperature were parameters that influenced the levels of activity of the promoters P_*pep0182 *_and P_*shp0182*_.

### Study of the binding of Rgg_0182 _protein of *S. thermophilus *upstream of *shp*_*0182 *_and *pep*_*0182 *_genes

The results above and the presence of the HTH motif on the N-terminal part of Rgg_0182 _protein suggested that Rgg_0182 _may regulate the transcriptional activities of the P_*shp0182 *_and the P_*pep0182 *_promoters by binding to their DNA sequences. To test this hypothesis, DNA electrophoretic mobility shift assay were carried out. To do so, the His_6_-Rgg_0182 _protein was overproduced in *E. coli *C41(DE3), verified by SDS-PAGE and Western blot (data not shown). Immobilized Metal ion Affinity Chromatography (IMAC) purification of the His_6_-Rgg_0182 _protein was performed. The purity of the Rgg_0182 _protein was assessed by SDS-PAGE using Coomassie blue protein staining, i.e. only one band of the expected molecular mass (35.7 kDa) was revealed (data not shown).

**Figure 4:**
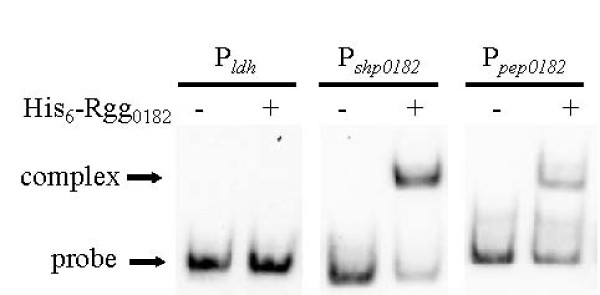
**Analysis of the Rgg**_**0182 **_**binding to DNA**. Electrophoretic mobility shift assay (EMSA) of the promoter regions of the two target genes (*shp*_*0182 *_and *pep*_*0182*_) of Rgg0182 in the absence or in the presence of the purified His_6_-Rgg_0182 _protein. DNA probes labelled with biotin (0.1 pmol each) were incubated with 2 pmol of Rgg_0182_. The P_*ldh *_probe is an *ldh *promoter fragment used as a negative control.

A 126 bp PCR amplified DNA fragment (Figure 1), including the entire 72 bp intergenic *rgg*_*0182*_-*shp*_*0182 *_region and part of the 5' end of the *shp*_*0182 *_and *rgg*_*0182 *_genes, was incubated with the purified His_6-_Rgg_0182 _protein. As can be seen in Figure 4, the Rgg_0182 _protein retarded the *shp*_*0182 *_promoter DNA fragment. The same experiment was realized with a 165 bp PCR amplified fragment, covering the entire 150 bp intergenic *rgg*_*0182*_-*pep*_*0182 *_region including the *pep*_*0182 *_promoter, and analogous results were obtained (Figure 4). The P_*ldh *_probe corresponding to the promoter region of the *ldh *gene was chosen as a negative control in EMSA experiments since its expression was not under the control of Rgg_0182_. Using P_*ldh *_as a probe, no DNA retardation was observed, demonstrating that Rgg_0182 _binds specifically to the promoter of its target genes. Thus, these results demonstrated conclusively that Rgg_0182 _activated the *shp*_*0182 *_and *pep*_*0182 *_genes transcription by binding to their promoter regions.

### Effects of the Rgg_0182 _protein on the transcription of genes encoding protease and chaperone proteins

**Figure 5:**
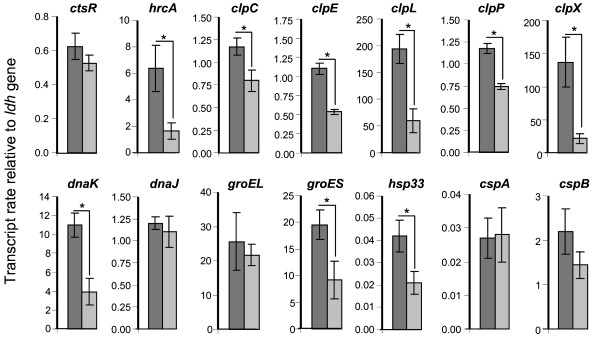
**Relative genes transcript level of *S. thermophilus *stationary phase cells grown in CDM medium at 30°C**. Total RNAs were extracted from stationary phase cells of *S. thermophilus *LMG18311 (dark gray bars) and its isogenic Δ*rgg*_*0182 *_mutant (light gray bars) grown in CDM at 30°C. Data are presented as the mean +/- standard deviation of the gene transcript levels measured from 3 independent experiments done in duplicate. Student's *t *test: *, p < 0.001.

The impact of temperature on the *rgg*_*0182 *_gene transcription suggested a role for the Rgg_0182 _protein on *S. thermophilus *LMG18311 adaptation to thermal changes. Thus, we hypothesized that Rgg_0182 _might control the transcription of genes encoding a set of heat- and cold-shock proteins including chaperones and proteases. Chaperones and ATP-dependent proteases play a major role for bacterial survival under conditions of heat stress where proteins tend to unfold and aggregate. Based upon the *S. thermophilus *LMG18311 genome sequence [26], genes predicted to encode the major chaperones and proteases involved in heat shock responses were selected for analysis: *clpC, dnaK, dnaJ, hsp33, groES, groEL, clpP, clpX, clpE, clpL *(Genbank Accession NC_006448, locus tags stu0077, stu0120-0121, stu0180, stu0203-0204, stu0356, stu0581, stu0602, stu1614, respectively). The two *cspA *and *cspB *genes (locus tags, stu0837-0838 respectively), involved in *S. thermophilus *cold stress response, were also included in this study. The transcript levels of these genes were measured by qPCR on stationary phase cells of the wild-type and the Δ*rgg*_*0182 *_mutant grown in CDM medium at 30°C (i.e. when *rgg*_*0182 *_was the most transcribed) from 3 independent experiments done in duplicate (Figure 5). In these conditions, the transcript level of almost all genes encoding protease and chaperone proteins (except that of *dnaJ, groEL, cspA *and *cspB*) was under-expressed in the Δ*rgg*_*0182 *_mutant compared to the wild type strain suggesting a role for Rgg_0182 _in the control of their transcription. The difference in the transcript abundance between the wild type and Δ*rgg*_*0182 *_mutant strains ranged from 1.5- to 20-fold and were statistically significant (P < 0.001). As described in other *Streptococcus *transcriptional analysis, a 1.5-fold difference in transcript level was interpreted as a significant difference in expression between the strains [21,23].

In low-GC Gram positive bacteria, the control of the transcription of the *clp *family genes and of *dnaK *and *groES *genes is primarily mediated by binding of the CtsR and HrcA repressors, respectively, to promoter region of target genes. In *S. thermophilus *LMG18311, we found CtsR operators (AGGTCAAANANAGGTCAAA) [6] upstream of *clpP, clpE, clpL, ctsR, clpC *and *groEL *genes and HrcA binding sites (GCACTC(N)_9_GAGTGCTAA) [30] only upstream of *hrcA, groEL *(with 2 mismatches) and *dnaJ *(6 mismatches). These results prompted us to evaluate the level of *ctsR *and *hrcA *transcripts (locus tags, stu0076 and stu0118 respectively) in the wild-type and the Δ*rgg*_*0182 *_mutant. These data revealed no significant difference for *ctsR *gene whereas the *hrcA *transcript level was nearly 4-fold reduced in the absence of *rgg*_*0182 *_suggesting that Rgg_0182 _positively controls *hrcA *transcription.

These results indicate that Rgg_0182 _is a positive transcriptional regulator of heat shock proteins encoding genes in particular of *hrcA, clpC, clpE, clpL, clpP, clpX, dnaK, groES *and *hsp33 *genes.

### Role of the *rgg*_*0182 *_gene in the heat shock response of *S. thermophilus*

Knowing that several *rgg *genes from pathogenic streptococci are involved in stress response and taking into account the above data, we checked whether *rgg*_*0182 *_could be involved in the *S. thermophilus *adaptation to heat shock. The heat tolerance was evaluated on stationary phase cells grown for 10 h in CDM medium (OD_600nm _= 1.5) or in LM17 medium (OD_600nm _= 2.5) at 30°C (where *rgg*_*0182 *_was found to be higher or lower transcribed, respectively) before (control condition) and after a 15, 30, 45 and 60 minutes incubation at 52°C (temperature limit for growth of *S. thermophilus *LMG18311 in our laboratory conditions). The experiments were realized 3 times independently in triplicate. Using the LM17 medium (data not shown), no significant difference was observed between the strains. An exposure at 52°C, whatever its duration, resulted in a 20% decrease of the survival of both strains.

**Figure 6:**
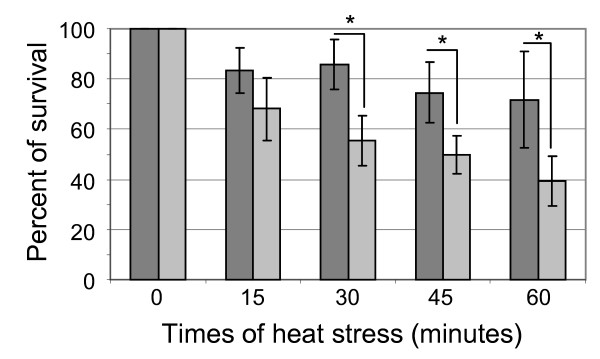
**Survival of the *S. thermophilus *strain LMG18311 and the Δ*rgg***_***0182 ***_**mutant after heat shock (0, 15, 30, 45 and 60 min at 52°C)**. *S. thermophilus *was cultivated in CDM medium at 30°C and then exposed to heat stress. The percentage of survival was calculated as *N*/*N*_0 _*×100 *where *N*_0 _is the CFU number of the control condition and *N *the CFU number in heat stress condition. Dark gray bars correspond to wild type strain and light gray bars correspond to Δ*rgg*_*0182 *_strain. Data are presented as the mean +/- standard deviation of 3 independent experiments done in triplicate. Student's *t *test: *, p < 0.001.

On the contrary, when stationary phase cells grown in CDM were exposed to a 52°C heat stress for up to 30 min, the mutant showed a significant increase of the sensibility compared to the wild type (p < 0.001) (Figure 6). The heat tolerance of the Δ*rgg*_*0182 *_mutant decreased gradually with the heat exposure time (72%, 53%, 46% and 38% of survival at 15, 30, 45 and 60 minutes, respectively). Between both strains, a difference of survival was observed at 30, 45 and 60 minutes where the mutant was up to 1.75 fold less resistant than the wild type strain. Thus, the decreased of survival of the mutant show that *rgg*_*0182 *_plays a role in *S. thermophilus *adaptation to heat stress.

### The Rgg_0182 _protein of *S. thermophilus *LMG18311 is involved in the transcription regulation of *clpE *and *cspB *genes in heat stress condition

**Figure 7:**
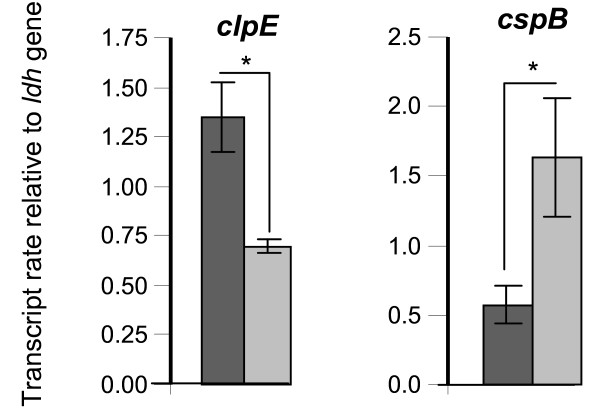
**Relative genes transcript level of *S. thermophilus *cells exposed to a heat stress**. Total RNAs were extracted from stationary phase cells of *S. thermophilus *LMG18311 (dark gray bars) and its isogenic Δ*rgg*_*0182 *_mutant (light gray bars) grown in CDM at 30°C until stationary phase and then exposed 30 min at 52°C (heat stress condition). Data are presented as the mean +/- standard deviation of the gene transcript levels measured from 3 independent experiments done in duplicate. Student's *t *test: *, p < 0.001.

The impairment of the survival of the Δ*rgg*_*0182 *_mutant cells following a sudden increase in temperature suggested that the *rgg*_*0182 *_gene may act to regulate the transcription of *S. thermophilus *genes involved in the heat shock response. To investigate a possible role for Rgg_0182 _in changes of the transcription of heat shock genes, the transcript level of genes encoding chaperones and proteases were measured by qPCR. The transcript levels of the 14 selected stress-responsive genes were studied, in three independent experiments done in duplicate, on stationary cells of the wild-type and the Δ*rgg*_*0182 *_mutant grown in CDM and exposed 30 minutes at 52°C. Our results showed that *clpE *and *cspB *genes were about 2-fold less and 3-fold more transcribed, respectively, in the mutant strain compared to wild-type (p < 0.001) (Figure 7). No significant difference was observed for the other genes studied (data not shown). This observation suggests that at high temperature, Rgg_0182 _is a positive regulator of *clpE *transcription and a negative regulator of *cspB *transcription.

## Discussion

The aim of the present study was to determine if Rgg_0182 _functioned as a transcriptional regulator. First, we showed that it was transcribed in a growth phase dependent manner i.e., in LM17 (at 30°C and 42°C) or CDM (at 42°C), a higher expression level was observed in exponential phase than in stationary phase. Interestingly, using CDM medium, it was found that the *rgg*_*0182 *_transcripts were more abundant at 30°C than at 42°C suggesting that *rgg*_*0182 *_transcription was also influenced by temperature. Because of their immediate vicinity with the *rgg*_*0182 *_gene, the transcription of *shp*_*0182 *_and *pep*_*0182 *_genes was hypothesized to be under the control of Rgg_0182_. This was confirmed by the use of transcriptional fusions showing that the activation of the P_*shp0182 *_and P_*pep0182 *_promoters required the presence of Rgg_0182 _and that their activity was optimal under the conditions were transcription of the *rgg*_*0182 *_gene was mostly expressed (i.e. in CDM medium at 30°C in stationary phase growth). Finally, to confirm the probable interaction of Rgg_0182 _with DNA, EMSA experiments were carried out and demonstrated conclusively that Rgg_0182 _binds to the promoter region of the *shp*_*0182 *_and *pep*_*0182 *_target genes. Together these results were in coherence with Rgg_0182 _being a transcriptional regulator, positively and directly, controlling the expression of *shp*_*0182 *_and *pep*_*0182 *_genes.

The *rgg*_*0182 *_locus combined a gene encoding a transcriptional regulator of the Rgg family with another gene encoding a small hydrophobic peptide of the SHP family. Recently, one of these *shp*/*rgg *loci, named *shp*/*rgg*_*1358 *_in LMD-9 has been demonstrated to encode two components of a novel QS mechanism [9]. This system involves a Rgg transcriptional regulator and a SHP pheromone that is detected and reimported into the cell by the Ami oligopeptide transporter. The target gene of the *shp*_*1358*_/*rgg*_*1358 *_pair, called *pep*_*1357C*_, is located just downstream of the *rgg*_*1358 *_gene, and encodes a secreted cyclic peptide [31]. By analogy with the Shp_1358_/Rgg_1358 _locus, we hypothesize that the SHP_0182_/Rgg_0182 _pair would also been involved in a QS mechanism with Shp_0182 _being a pheromone possibly controlling the activation of the Rgg_0182_. Thus, one hypothesis to explain the high *rgg*_*0182 *_transcript level in CDM is to consider that the *rgg*_*0182 *_transcription would be under the control of the Rgg_0182_/SHP_0182 _pair and to consider that in a medium free of peptide, the internalization of *shp*_*0182 *_by Ami transporter would be facilitated. Moreover, the mechanism of *rgg*_*0182 *_expression seemed to be more complex than that of *rgg*_*1358 *_since not only influenced by the culture medium but also by the temperature. Further experiments will be done (i) to determine whether the QS mechanism involving the SHP_1358 _and the Rgg_1358 _can be generalized to other SHP/Rgg pairs, including SHP_0182_/Rgg_0182 _pair and (ii) to understand the mechanism by which temperature could influence the *rgg*_0182 _expression.

On the other hands, induction of the *rgg*_*0182 *_expression at 30°C suggests that this gene might participate in the physiological adaptation of *S. thermophilus *to this temperature. When cells were cultivated in CDM at 30°C, the inactivation of *rgg*_*0182 *_was associated with a reduce expression of genes encoding chaperone and protease proteins. In *Bacillus subtilis*, the DnaKJ complex facilitates substrates folding to the native state and the GroESL complex provides an isolated environment for the proper folding of small protein substrates [32]. The degradation of unfolded proteins and small peptides is ensured by a protease complex composed of the protease subunit ClpP and several ATPases of the Clp family [32]. Thus, the Rgg_0182 _is a transcriptional regulator whose biological roles would be to control the homeostasis of chaperone and protease proteins in cells grown at 30°C in CDM. This is in concordance with data obtained in *S. pyogenes *where Rgg is found (at the protein level) to control the expression of ClpL, ClpP, GroEL and DnaK in stationary phase (4). Furthermore, it was shown that ClpL protein of *S. thermophilus *Sfi39 is necessary for correct response to both heat and cold stresses [4].

Results of qPCR experiments also showed an effect of Rgg0182 on *hrcA *expression. However, preliminary EMSA results (data not shown) indicated that the Rgg0182 protein did not bind to the *hrcA *promoter region. This suggests that the transcription of *hrcA *obviously is stimulated by Rgg_0182 _indirectly, perhaps by influencing the expression of another regulatory protein. Such indirect regulation has already been reported for other Rgg proteins [12,13,21] and, in the present study, might be extended to, at least, some of the *rgg*_*0182 *_distal target genes.

Finally, to assess the significance of Rgg-associated changes in the expression of genes involved in the heat shock response, we checked whether the deletion of *rgg*_*0182 *_had an impact on the survival of the strains under heat stress conditions (shift from 30°C to 52°C for 15 min to 60 min). Interestingly, an impaired survival of the mutant was observed but only when the cells were cultivated in the CDM medium, i.e. in conditions where the difference in the level of *rgg*_*0182 *_transcripts was maximal between both strains. In the mutant cultivated in CDM, the percent of survival decreased with the duration of the heat exposure. These results demonstrated a role for Rgg_0182 _in *S. thermophilus *fitness in response to sudden increased of the temperature. As observed in other streptococcal strains [24,25], the deletion of the *rgg*_*0182 *_gene is not associated with a drastic modification of the survival to stress suggesting that this regulator is not essential but important for heat stress adaptation. Furthermore, our results showed that *cspB *and *clpE *genes were 2-fold lower and 3-fold higher, respectively, in the mutant compared to the wild-type strain after the heat stress. Data from literature indicate that most Csp proteins are required when cells are grown at low growth temperature [2,3]. Thus, the Rgg_0182 _would negatively control the production of CspB when the latter is not required. Moreover, in *S. pneumoniae*, the *clpE *gene has been demonstrated to be required for thermo-tolerance [33], therefore we hypothesize that the heat sensitivity of the *S. thermophilus *Δ*rgg*_*0182 *_mutant would result, at least partially, from a reduced level of ClpE expression. Alternatively, it is also conceivable that Rgg_0182 _regulates the transcription of other genes encoding proteins involved in the *S. thermophilus *heat stress response. A transcriptomic analysis would identify all targets of this regulator within *S. thermophilus *LMG18311.

## Conclusions

In conclusion, our study gave a better understanding of the thermal adaptation of the important dairy starter, *S. thermophilus*. These data showed the importance of the Rgg_0182 _transcriptional regulator on the survival of *S. thermophilus *during industrial processes and more specifically during changes in temperature.

## Methods

### Bacterial strains, media and reagents

*Streptococcus thermophilus *LMG18311 and its derivatives are presented in Table 1. *S. thermophilus *strains were grown at 30 or 42°C in M17 medium with lactose (10 g/l) (LM17, a classical medium for *S. thermophilus *growth) [34] or in a chemically defined medium (CDM, a peptide free-medium) [35]. Pre-cultures were incubated at 42°C in milk medium except for the luciferase assays as mentioned below. For numeration, agar was added to the medium (15 g/l) and cells were incubated under anaerobic conditions using GENbox anaer in Generbox jars (bioMérieux SA, Marcy-l'Etoile, France). *S. thermophilus *strains containing the pG^+^host9 vector [36] were cultivated in the presence of erythromycin (final concentration 2 μg/ml) at 30°C when plasmid self-maintenance was required and at 42°C for selection of clones with the chromosome's integrated plasmid.

**Table 1:** Bacterial strains and plasmids used in this study

Strains and plasmids	Genotype/phenotype/source	Origin or reference
***Streptococcus thermophilus***		

LMG18311	Wild-type; isolated from yogurt.	BCCM LMG
Δ*rgg*_*0182*_	Derivative of LMG18311 carrying a 699 bp deletion in the *rgg*_*0182 *_gene	This study
LMG18311 *blp*::P_*pep0182*_*-luxAB*	LMG18311 containing a P_*pep0182-luxAB *_fusion at the *blp *locus	This study
LMG18311 *blp*::P_*shp0182*_*-luxAB*	LMG18311 containing a P_*shp0182-luxAB *_fusion at the *blp *locus	This study
Δ*rgg*_*0182 *_*blp*::P_*pep0182*_*-luxAB*	Δ*rgg*_*0182 *_containing a P_*pep0182-luxAB *_fusion at the *blp *locus	This study
Δ*rgg*_*0182 *_*blp*::P_*shp0182*_*-luxAB*	Δ*rgg*_*0182 *_containing a P_*shp0182-luxAB *_fusion at the *blp *locus	This study
		
***Escherichia coli***		

EC101	*supE hsd-5 thi *Δ*(lac-proAB) *F'*(traD6 proAB*^*+ *^*lacI*^*q *^*lacZ*Δ*M15) repA*^*+*^, derivative of TG1 strain	[37,38]
EC101 pG9Δ*rgg*_*0182*_	Derivative of EC101 containing pG9Δ*rgg*_*0182*_	This study
C41(DE3)	Derived from BL21(DE3) (F^- ^*ompT hsdS*_B_(r_B_^- ^m_B_^-^) *gal dcm met *(DE3); Novagen); at least one uncharacterized mutation	[39]
C41(DE3) pET15b::*rgg*_*0182*_	Derivative of C41(DE3) containing pET15b::*rgg*_*0182*_	This study
		
**Plasmid**		

pG^+^host9	Thermosensitive plasmid replication origin from pVE6002, used for gene replacement; Ery^R^	[36]
pG9Δ*rgg*_*0182*_	Derivative of pG^+^host9 carrying a 699 bp (from nucleotide 82 to 783) deletion for *rgg*_0182_	This study
pGICB004	Thermosensitive plasmid, *Em*^*r*^; used for the construct of transcriptional fusions. Allow the integration of the *luxAB *genes of *Photorabdus luminescens *at the *blp *locus of the *S. thermophilus *chromosome	[9]
pGICB004::P_*pep0182*_	Derivative of pGICB004 used to introduce a P_*pep0182*_*-luxAB *transcriptional fusion at the *blp *locus	This study
pGICB004::P_*shp0182*_	Derivative of pGICB004 used to introduce a P_*shp0182*_*-luxAB *transcriptional fusion at the *blp *locus	This study
pET15b	Expression vector for N-terminal His_6_-tagged fusion; Amp^R^	Novagen
pET15b::*rgg*_*0182*_	Derivative of pET15b carrying a 864 bp insert coding a Rgg_0182 _His_6_-tagged protein	This study

BCCM Belgian Coordinated Collections of Microorganisms, LMG Laboratory of Microbiology and Genetics, University of Gent

*E. coli *strains were grown in Luria-Bertani (LB) medium [37] at 30 or 37°C with shaking (250 rpm). *E. coli *EC101 [38] was used as a host for recombinant plasmids derived from pG^+^host9 [36] and *E. coli *C41(DE3) [39] for recombinant plasmids derived from pET15b (Novagen). When required, erythromycin (150 μg/ml) or ampicillin (150 μg/ml) was added.

### DNA and RNA manipulations

Conventional techniques for DNA manipulation, such as preparation of chromosomal and plasmidic DNA, restriction enzymes digests, PCR experiments, transformation by electroporation and Southern blotting were performed as described [37]. Plasmids and primers used in this study are listed in Table 1 and Table 2, respectively. Oligonucleotides were purchased from Eurogentec (Liège, Belgique). Sequencing reactions were done from PCR products by the Beckman Coulter Genomics Society (Grenoble, France) using an ABI 3730XL Sanger sequencing platform.

**Table 2:** Primers used for PCR and qPCR

Name		**Primer sequence (5'**-**3')**	
**Primers for vectors construction**			

Rgg_0182_I1		CTGGAA**CTGCAG**GAGCAGC	
Rgg_0182_I2		ATAATTTGA**GAATTC**TGTACCTT	
Rgg_0182_II1		TCTCTG**GAATTC**TTTAAATTG C	
Rgg_0182_II2		CTTGTC**CTGCAG **TCTCACTCCC	
Rgg_0182_SE1		CCCCCCC**CATATG**GGAAAACAAAATGAACTG	
Rgg_0182_SE2		CCCC**GGATCC**TTAACCTACAATCGACTTAAA	
Pep0182up		GAA**GAATTC**GTAAAAAGCATCAGATTTTAC	
Pep0182down		AACT**ACTAGT**GCATTTTAAGTCGATTGTAGG	
SHP0182up		GAA**GAATTC**AACAATTTCATATGATTTCCTCC	
SHP0182down		AACT**ACTAGT**TCAGTCTCTCCTCTTTC	
			
**Primers for DNA EMSA**			

Pldh-5'		ACGCTTTCACTTAATAATTC	
Pldh-3'		TGGTCTAAACATCTCCTTA	
Pshp-5'		GTAAATACATGTCAATAGGAC	
Pshp-3'		TTTGTTTTCCCATATATGCAACC	
Ppep-5'		AGCATCAGATTTTACTCCAGATG	
Ppep-3'		TTGTAGGTTAATCCCGTTTATGC	
			
**Primers for gene expression analysis**			

**Gene**	**Locus**	**Forward**	**Reverse**
*ldh*	stu1280	AAGCTATCCTTGACGATGAA	AATAGCAGGTTGACCGATAA
*rgg*_*0182*_	stu0182	GAAGTGGAGGAGTTGCCTAA	CCCAGCTCTCAATCCCAAA
*hrcA*	stu0118	ACACCTCTTCAAGGAACTGAT	GTCACTTCATCATCGGAGATA
*dnaK*	stu0120	GACATTGACGAAGTCATCCT	GCACCCATAGCAACTACTTC
*dnaJ*	stu0121	CGTGAAGTGACATGTAAGACA	ACCAAGTGGTGTTTGTGTATC
*groES*	stu0203	GAAGGTACCCGTACTCTTACTG	AACGTAATCTTCTCCGTCTTTA
*groEL*	stu0204	ATTGCTTATAATGCCGGTTA	AGCGTTAAATCCTGTACCAA
*hsp33*	stu0180	TTTAGTAGGTCCTTTCATG	CGATTTCACCAGAAATAAGC
*cspA*	stu0837	ATTGGTTTAACGCTGACAAAG	TAACCTTTTGACCTTCATCGT
*cspB*	stu0838	TATGGCAAATGGAACAGTAAA	CAAGTGATTTGAATCCATCAG
*ctsR*	stu0076	AGATCAGCTCAGCGAACA	AGAATACGCGAACGAATG
*clpC*	stu0077	ACTGGCAGATTATACCAAAGAC	CACCTACCAAGACAGGATTATT
*clpP*	stu0356	CTTGCTCAAGACTCGTAATAACT	AAGCCATATTCAAGTGTTTCTT
*clpX*	stu0581	TGGACTTATCCCTGAATTTATC	AAGACAAGAGGGTTTGATACTG
*clpE*	stu0602	CCGTACCAAGAACAATCCT	ACGGATAACTTGCTTGCTT
*clpL*	stu1614	CGTTTCGACGCAGTTATT	TTCGCTAACTGCCAAGTC

Restriction sites are indicated with bold letters: *Pst*I: **CTGCAG**; *Eco*RI: **GAATTC**; *Spe*I: **ACTAGT**; *Nde*I: **CATATG**; *Bam*HI: **GGATCC**

### Plasmids and strains construction

The Δ*rgg*_*0182 *_mutant was constructed following the experimental procedure described by Layec *et al*. (2009) [40] using the following primers pairs: rgg_0182_I1/rgg_0182_I2 and rgg_0182_II1/rgg_0182_II2 to amplify fragments I and II, respectively. The resulting Δ*rgg*_*0182 *_mutant was checked by sequencing.

The construction of pGICB004::P_*pep0182 *_and pGICB004::P_*shp0182 *_vectors was done as described in [9]. Briefly, the *pep*_*0182 *_and *shp*_*0182 *_promoters were amplified by PCR using the couple of oligonucleotides Pep0182up/Pep0182down and SHP0182up/SHP0182down, respectively. Following a digestion with restriction enzymes *Spe*I/*Eco*RI, both fragments were independently inserted downstream from *luxAB *in the pGICB004 plasmid previously digested with the same restriction enzymes. The pGICB004 plasmid was used since it allows the integration of transcriptional fusions to the *luxAB *reporter genes at the *blp *locus in *S. thermophilus *[9]. The final pGICB004::P_*pep0182 *_and pGICB004::P_*shp0182 *_plasmids were used to transform the LMG18311 and in its derivative Δ*rgg*_*0182 *_strain. The strains LMG18311 *blp*::P_*pep0182*_*-luxAB*, LMG18311 *blp*::P_*shp0182*_*-luxAB*, Δ*rgg*_*182 *_*blp*::P_*pep0182*_*-luxAB *and Δ*rgg*_*182 *_*blp*::P_*shp0182*_*-luxAB *were obtained by gene replacement at the *blp *locus as described in [9].

For the construction of the pET15b::*rgg*_*0182 *_plasmid necessary for the Rgg_0182_-His_6_-tagged protein overexpression, the *rgg*_*0182 *_(864 bp) gene was amplified by PCR using the primers Rgg_0182_SE1 and Rgg_0182_SE2 which contained *Nde*I and *Bam*HI restriction sites, respectively, with *S. thermophilus *LMG18311 chromosomal DNA as template. The amplified DNA fragment was double digested with restriction enzymes *Nde*I/*Bam*HI and fused in-frame to the 3'end His_6_-tagged of pET15b vector (Novagen), generating the pET15b::*rgg*_*0182 *_plasmid. The in-frame fusion was confirmed by DNA sequencing.

#### Luciferase assays

To perform luciferase assays, pre-cultures were grown overnight at 30 or 42°C in CDM or LM17 medium. Pre-cultures were then diluted to an OD_600nm _of 0.05, in 50 ml of respective appropriate medium and temperature. A volume of 1 ml of the culture was sampled at regular intervals during the growth until the stationary phase and analyzed as follows: OD_600nm _was measured, 10 μL of a 0.1% nonyl-aldehyde solution was added to the sample and the luminescence was measured with a Luminoskan TL (Labsystems). Results are reported in relative luminescence divided by the OD_600nm _(AU). Three independent experiments were realized.

### Overexpression, purification of Rgg_0182_-His_6_-tagged protein and Western blotting

Expression of the His_6_-tagged protein was induced in *E. coli *C41(DE3) containing pET15b::*rgg*_*0182 *_for 4h at 30°C by adding Isopropyl *β*, D-thiogalactopyranoside (IPTG, 1mM final concentration) to the OD_600nm _= 0.5 culture. Cells were harvested by centrifugation at 14,000 rpm, at 4°C for 30 min. The supernatant was discarded and cells were suspended in lysis buffer (50 mM phosphate sodium pH 8.0, 300 mM NaCl, and 10 mM imidazol) and stored at -20°C. The cells were disrupted on ice with a microtip of Sonifier 250 (Branson Ultrasonics). The soluble fraction including the recombinant His_6_-tagged protein was collected by centrifugation at 20,000 rpm for 45 min at 4°C and loaded on an affinity chromatography column equilibrated with lysis buffer. When the UV absorbance at 280 nm had fallen to the zero baseline, the recombinant Rgg_0182 _protein was eluted by elution buffer (50 mM phosphate sodium pH 8.0, 300 mM NaCl, 250 mM imidazol). The eluted fraction was collected and finally concentrated in Tris EDTA buffer pH 8.0. The purity of the His_6_-tagged proteins was confirmed by sodium dodecyl sulphate-polyacrylamide gel electrophoresis (SDS-PAGE) using 15% acrylamide resolving.

For Western blot experiments, proteins were size separated by SDS-PAGE 12% acrylamide resolving gel and electroblotted onto polyvinylidene difloride (PVDF) membrane (Roche Applied Science) using a semi-dry blotting system (Bio-Rad). After transfer, the PVDF membrane was blocked with 5% skim milk in Tris-buffered saline containing 0.1% tween 20 (TBS-T) for 1 h. The membrane was subsequently incubated for 1 h with penta-His antibodies (1:10,000) (Qiagen), washed three times with TBS-T and incubated for 1 h with conjugated goat anti-mouse immunoglobulin G (H + L)-horseradish peroxidase (1:10,000) (Bio-Rad). The membrane was washed three times with TBS-T. Finally, the antibodies-antigen complexes were visualized by using the Bio-Rad Immun-Star Western system as described by the manufacturer and were detected by chemiluminescence, using the Chemi-Doc from Bio-Rad.

### DNA electrophoretic mobility shift assay (EMSA)

The DNA binding of the His6-tagged Rgg_0182 _protein to the *shp*_*0182 *_and *pep*_*0182 *_promoter regions was tested by EMSA using the LightShift Chemiluminescent EMSA Kit (Thermo Scientific). The promoter regions of *ldh *(P_*ldh*_, 110pb), *shp*_*0182 *_(P_*shp0182*_, 126 bp) and *pep*_*0182 *_(P_*pep0182*_, 165 bp) were amplified by PCR using the Pldh-5'/Pldh-3', Pshp-3'/Pshp-5' and Ppep-3'/Ppep-5' primers, respectively. These were 3'-end biotin labelled with Biotin 3' End DNA Labeling Kit (Thermo Scientific) and used in EMSA according to the manufacturer's instructions. Chemiluminescent detection of biotin DNA on membranes was realised with the Chemi-Doc apparatus (Bio-Rad).

### RNA extraction and quantitative RT-PCR (qPCR) experiments

RNA extractions were adapted from Kieser *et al*. (1999) [41]. RNAs were extracted from cultures grown in CDM or LM17 medium in exponential, transition, or stationary phase at 30 or 42°C. RNAs were also extracted from stationary phase cells exposed to a 30 min temperature shift from 30 to 52°C. The RNAs were treated with amplification grade DNase I (Euromedex). The quantity and quality of the RNA samples were verified by agarose gel electrophoresis and by measuring their absorbance at 260 and 280 nm (NanoDrop-1000). Reverse transcription was performed according to the manufacturer's instructions (MMLV-reverse transcriptase, Invitrogen). cDNA was generated from 1.25 μg of DNA-free RNA and used for qPCR analysis of transcription of *rgg*_0182 _gene and its potential target genes transcript levels. Gene transcripts quantification was done using the CFX96 manager software (Bio-Rad) with the following program: 1 cycle at 98°C for 3 min and 40 cycles at 95°C for 10 s and at 58°C for 45 s. The amplification reactions were carried out with SYBR Green Supermix (Bio-Rad). Melting curve analysis was performed with 0.5°C increments every 10 s from 55 to 95°C to check that the cDNA amplification did not lead to secondary products. The primers used for qPCR are listed in Table 2. The efficiency of all primers pairs was checked in qPCR using serial dilutions of cDNA, and ranged from 90 to 100%. The level of gene transcript was calculated with *ldh *gene as the internal control gene for normalization [23].

### Physiological characterization of the Δ*rgg*_*0182 *_mutant

Stationary phase cells were harvested from cultures grown in CDM at 30°C by centrifugation at 4,500 rpm for 10 min. Cells were washed twice and resuspended in 10 mM sterile phosphate buffer, pH 7.0 with a final OD_600nm _of 1.0. Then, for heat stress, cells were treated by incubation at 52°C during 15, 30, 45 and 60 min (heat stress condition) or not (control condition). Cultures were then diluted to appropriate concentrations, spread on LM17 agar plates and incubated overnight at 42°C under anaerobic conditions. A percentage of survival was calculated as *N*/*N*_0 _*×100 *where *N*_0 _is the CFU number of the control condition and *N *the CFU number after heat stress condition. Three independent experiments done in triplicate were realized.

### Statistical analysis

Data are expressed as mean +/- standard deviation (SD). Statistical analysis was performed with Student's *t *test. A *p *value < 0.05 was considered statistically different.

### Nucleotide sequence accession number

The DNA sequence reported in this paper has been deposited in GenBank under accession number JF699754.

## Authors' contributions

Conceived and designed the experiments: RH EB BD NL. Performed the experiments: RH EB RG SB BF. Analyzed the data: RH EB RG BF NL. Wrote the paper: RH EB NL. All authors read and approved the final manuscript.
